# Identifying hypothetical genetic influences on complex disease phenotypes

**DOI:** 10.1186/1471-2105-10-S2-S13

**Published:** 2009-02-05

**Authors:** Benjamin J Keller, Richard C McEachin

**Affiliations:** 1 Eastern Michigan University, Computer Science Department, Ypsilanti, MI 48197, USA; 2 Department of Psychiatry, University of Michigan, Ann Arbor, MI 48109, USA; 3 National Center for Integrative Biomedical Informatics, Ann Arbor, MI 48109, USA

## Abstract

**Background:**

Statistical interactions between disease-associated loci of complex genetic diseases suggest that genes from these regions are involved in a common mechanism impacting, or impacted by, the disease. The computational problem we address is to discover relationships among genes from these interacting regions that may explain the observed statistical interaction and the role of these genes in the disease phenotype.

**Results:**

We describe a heuristic algorithm for generating hypothetical gene relationships from loci associated with a complex disease phenotype. This approach, called Prioritizing Disease Genes by Analysis of Common Elements (PDG-ACE), mines biomedical keywords from text descriptions of genes and uses them to relate genes close to disease-associated loci. A keyword common to, and significantly over-represented in, a pair of gene descriptions may represent a preliminary hypothesis about the biological relationship between the genes, and suggest the role the genes play in the disease phenotype.

**Conclusion:**

Our experimentation shows that the approach finds previously published relationships, while failing to find relationships that don't exist. The results also indicate that the approach is robust to differences in keyword vocabulary. We outline a brief case study in which results from a recently published Type 2 Diabetes association study are used to identify potential hypotheses.

## Background

**Figure 1:**
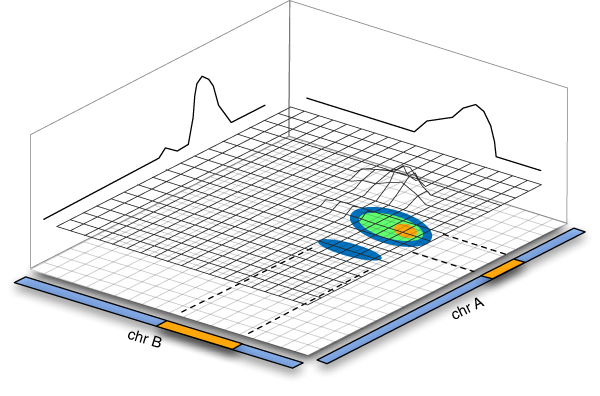
**Interacting linkage peaks**. Linkage peaks with statistical interaction suggest pairs of regions of the genome in which genes that co-contribute to a disease may be found.

In the study of the genetics of complex diseases such as Bipolar Disorder, we see statistical interactions between disease-associated loci such as the interacting linkage peaks depicted in Figure 1, or interactions between pairs of SNPs in a genome-wide association study. These observations suggest that one or more genes from these interacting loci are somehow involved in a common mechanism that impacts the disease. To better understand the disease, we want to discover relationships among the blocks of genes implied by the interacting loci that explain the statistical interaction and the role of the genes in the disease. We consider this task as one of finding hypothetical genetic influences on the disease phenotype, and approach the problem by finding biomedical keywords common to Entrez Gene [1] descriptions of pairs of genes from the interacting regions. Each such keyword relates the gene pair, and may lead to a novel hypothesis about how the genes contribute to the disease phenotype.

Other candidate-gene finding tools use similar strategies (see the survey by Oti and Bruner [2]), but the majority of these approaches use some form of formal annotation (e.g., GO terms) instead of text features. For instance, POCUS [3] uses GO terms together with InterPro domains to find candidate gene interactions; Endeavour [4] and NARADA [5] use common GO terms to define gene networks; and BITOLA [6] uses MeSH terms as concepts that are related to genes by co-occurrence. Other tools that use text mining, such as PDQ Wizard [7] use co-occurrence of genes in the literature to infer relationships, which provides different information than our approach. We believe that our approach of mining unstructured gene descriptions for keywords is novel, and complementary to these other approaches.

## Results

This paper describes our strategy and its implementation in a tool called PDG-ACE (Prioritizing Disease Genes by Analysis of Common Elements). Here, we discuss how Entrez Gene records are mined, and describe the algorithm and statistical tests. We describe validation and parameter tuning experiments, as well as a case study using the genes identified in a recent Type 2 Diabetes (T2D) study [8].

### Mining gene descriptions

The PDG-ACE algorithm uses an association of keywords with genes mined from Entrez Gene records. We have developed tools that build these associations in two ways: matching Entrez Gene text against a dictionary of keywords, and naïve recognition of phrases within the text. The first method finds all longest full matches to the dictionary. The second finds the longest non-stopword phrases within the text. In both cases, stopwords are filtered out, using a stopword list consisting of common English words.

**Figure 2:**
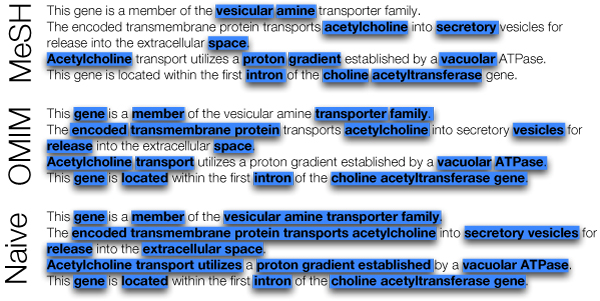
**Differences in vocabulary**. The differences among the three vocabularies are illustrated for the Entrez Gene description of SLC18A3.

We constructed three vocabularies. For each, we first derived an initial vocabulary, and then filtered the keywords to keep only those that are rare in Entrez Gene records. The first vocabulary is based on Medical Subject Headings (MeSH), from which we created a vocabulary by splitting headings to make phrases likely to be seen in text. We created the second vocabulary, meant to eliminate bias due to a particular dictionary, by extracting naïve keyphrases directly from Entrez Gene records. The third vocabulary was created to emphasize keywords related to neurological disorders. To do this, we extracted naïve keyphrases from OMIM [9] records containing the substring "neuro". Figure 2 illustrates the differences among the three vocabularies, which we refer to as the MeSH, NAÏVE and OMIM vocabularies.

Once the initial association is mined, we screen the vocabulary to eliminate keywords that are very rare or very common in Entrez Gene records. Keywords with fewer than three occurrences are eliminated. The threshold for eliminating common keywords uses an approximation to the statistical significance test used in the algorithm. Letting *G *be the total number of genes, and *N *be the total number of keywords, and assuming a Bonferroni correction of 0.05/*N*, we want keywords with at most $\sqrt{0.05\cdot G^{2}/N}$ occurrences. This narrows the vocabulary to words that are likely to be common across gene pairs and also pass the significance test for over-representation.

Our association-building tools are able to mine from different text elements of the Entrez Gene records. For the MeSH and OMIM vocabularies, we mined the official full name (gene-ref_desc), aliases (gene-ref_syn_E), summary (Entrezgene_summary), annotation from other databases (other-source_anchor), and Gene RIF (gene-commentary_text) elements. For the NAÏVE vocabulary we did not mine the synonyms and other sources, because of the large number of unique terms. Note that in preprocessing we build a list of genes and their locations from an authoritative source. Results presented here are based on hg18 data tables from the UCSC genome browser [10]. Genes are also filtered to include only current Entrez Gene records.

### Algorithm

The primary input to PDG-ACE is a pair of disease-associated loci and a delta in basepairs from each locus. These inputs define a pair of chromosomal regions from which genes are considered. The algorithm does one run using this observed pair of disease-associated loci, then performs permutations to determine the significance of the observed results.

In each run, each keyword is scored with the number of possible pairs of genes, across the loci, that the keyword describes. All keywords common to at least one gene in each region will have a nonzero score. The observation run assigns a score to each keyword at the observed interacting locus pair, and keywords that have a zero score are filtered prior to the permutation runs. The permutations are run on blocks consisting of the same number of sequential genes as the observed loci. A block is selected by randomly choosing a chromosome arm then randomly picking a block of sequential genes on that arm. If the arm is too small, then another arm is chosen until one that has enough genes is found.

As permutations are run, the rank of each observed keyword score is determined. If, on completion of the permutation runs, the score of a keyword ranks above a user provided threshold, the keyword, its rank, and the corresponding genes from both loci are reported. The *p*-value for a keyword is the proportion of scores for permutation runs that are greater than or equal to the observation run score. In post-processing, a Bonferroni correction can be applied so the threshold for significance is 0.05/*N*, where *N *is the number of keywords in the vocabulary.

### Validation testing

We validated our approach using published studies as positive controls and randomly selected locus pairs as negative controls. Two control studies used microsatellite markers as loci, and the rest used genes.

For validation, the positive controls were from seven published studies showing statistically significant gene-gene interactions. These include two breast cancer studies [11,12], and studies of osteoporosis [13], anorexia nervosa [14], colorectal cancer [15], asthma [16], and neural tube defects [17]. Each of these studies found statistical evidence of gene-gene interactions. Our expectation was that PDG-ACE would find keywords that are over-represented and consistent with genetic interactions predisposing these diseases. The negative controls were pairs of randomly selected genes from Entrez Gene, with the expectation that PDG-ACE would not find over-represented common keywords.

For each locus pair, we tested loci defined by deltas from 10^3 ^basepairs (KBP) to 10^6 ^basepairs (MBP) from each gene's transcription start site. At each delta, we ran PDG-ACE in duplicate, and performed trials to ensure a sufficient sample as described below. Tests were performed in parallel, using all three vocabularies (OMIM, MeSH, and NAÏVE). In all but one case, results for deltas greater than 500 KBP showed no significant keywords; we report only smaller regions.

Several trials may be needed to determine the number of permutations at which the sample of the genome yields a consistent measure of significance for rare keywords. Each test is run in duplicate starting with one million iterations. The sample is considered sufficient if the top three keywords are identical, and in the same order in both runs. If that criterion is not met, we increase the number of permutations and re-run the test in duplicate until the criterion is met.

Table 1 shows hits for the positive controls and Table 2 shows hits for the negative controls, both using the MeSH vocabulary of 2531 keywords. Note that the pattern of hits in the positive controls is significantly different from the negative controls (χ^2 ^*p*-value < 0.01). In general, the strongest evidence for multi-gene effects is near the observed loci (+/-1 KBP), and the pattern of hits is consistent with *p*-values from the control studies. As expected, in most, but not all, cases, significantly over-represented, common keywords are consistent with disease etiology. For example, in the first breast cancer study, the *COMT*-*CCND1 *genetic interaction is significant (*p*-value 0.014 in the interaction study) and the over-represented, common keyword is "estradiol" (*p*-value 0.041). "Estradiol" is used in the same context at both loci, and may offer insight into hormone sensitive breast cancer etiology.

**Table 1:** Validation results for positive controls. Results of validation experiments on positive controls from previous genetic studies. The *p*-values are from the original study, and the numeric column labels refer to the delta from the loci in KBP.

**Phenotype**	**Locus**	**Locus**	**P-Value**	**1**	**100**	**250**	**500**
Breast Cancer^7^	XPD	IL10	0.007	✔	✔	✔	✔
Breast Cancer^7^	GSTP1	COMT	0.007	✔	✔		
Breast Cancer^7^	COMT	CCND1	0.014	✔			
Breast Cancer^7^	BARD1	XPD	0.014		✔		
Breast Cancer^7^	CYP17	GADD45g	0.062				
Breast Cancer^7^	TNFa	p27	0.079	✔			
Breast Cancer^7^	BARD1	ESR1	N/A				
Breast Cancer^7^	BARD1	p27	N/A				
Breast Cancer^8^	GSTM1	CYP2e1	0.05	✔	✔	✔	
Osteoporosis^9^	NR3C1	ESR2	0.047	✔			
Osteoporosis^9^	NR3C1	HDC	N/A				
Osteoporosis^9^	RANK	TNFR2	N/A		✔		
Anorexia Nervosa^10^	MAOA	SLC6A2	0.019	✔	✔	✔	✔
Colorectal Cancer^11^	ALDH2	ADH1B	0.001	✔	✔	✔	✔
Asthma^12^	CD14	IL4Ra	0.001	✔			
Neural Tube Defect^13^	CbetaS	MTHFR	0.007	✔	✔	✔	
Neural Tube Defect^13^	MTRR	MTHFR	0.003	✔	✔		✔
Neural Tube Defect^13^	MTRR	FOLH1	0.004	✔			

In two cases, gene families provide the strongest evidence at a locus pair. For the *BARD1*-*XPD *(a.k.a. *ERCC2*) interaction in the first breast cancer study (*p*-value 0.014), *BARD1 *as well as paralogs *ERCC2 *and *ERCC1 *refer to keyword "dna repair" (*p*-value 0.009). Since *ERCC2 *and *ERCC1 *are adjacent in the genome, evidence of the multi-gene effect extends beyond the bounds of the *XPD *gene, out to +/-100 KBP. Arguably, cancer-related effects of variations in *ERCC2 *may be influenced by variations in *ERCC1*, so both of the *ERCC *genes should be evaluated for genetic variation related to breast cancer. A similar effect is seen for *RANK *(a.k.a. *TNFRSF11*)-*TNFR2 *(a.k.a *TNFRSF1B*) in the osteoporosis study, where *TNFRSF1B *and *TNFRSF8 *are adjacent in the genome. The authors of the previous study did not find significant evidence for a genetic interaction. However, all three genes refer to "marrow" (corrected *p*-value 0.033), consistent with bone disease, so the true genetic interaction may have been hidden in the previous study, but revealed by PDG-ACE. In both the breast cancer and osteoporosis studies, evidence is consistent with gene family effects on the phenotype, as expected in complex diseases.

These validation experiments show that findings from PDG-ACE are generally consistent with the strength of prior evidence, as seen by comparing *p*-values found in the interaction analyses and the pattern of significant keywords found by PDG-ACE. In general, evidence of commonality falls off as delta grows larger. This observation coincides with the experiments for the two interaction studies [18,19] based on variation in microsatellite markers. Results of these experiments (not shown) indicate that PDG-ACE is not effective for this type of prior information. Negative controls generally show no evidence of common effects, as expected (Table 2).

**Table 2:** Validation results for negative controls. Results of validation experiments on negative controls of randomly selected gene pairs.

**Locus**	**Locus**	**1**	**100**	**250**	**500**
ATG4C	TBX21				
HLA-C	CYP27B1				
ITGAM	GNPTAB				
MBD4	ATP4A				
PPIE	FBXO17				
SEPW1	USP9X				
SERPINA13	BCL3				✔
VKORC1	FUT1				
CFHR1	ATP6V0A1				
GCSH	SRPK2				
CCDC64	MNAT1			✔	
HRAS	PRNPIP				

We also did experiments to study the impact of choosing a particular vocabulary by repeating the positive control experiments using each of the three vocabularies (MeSH, OMIM, and NAÏVE). We ran the experiments in triplicate, using identical parameter settings for each of the vocabularies. Table 3 shows the results from these experiments. Interestingly, the pattern of hits is quite similar for all three vocabularies, even though the specific keywords in the vocabularies are different. For example, for the *GSTM1*-*CYP2e1 *locus pair at 1 KBP in the second breast cancer study, the common over-represented keywords for the MeSH vocabulary are: "cyp2e1", "ethanol", "smoke", "area", "stomach"', "toxicity", and "xenobiotics". For the NAÏVE vocabulary the corresponding list is: "alcoholics", "cigarette smoke", "high-risk area", "stomach cancer", "incomplete intestinal metaplasia", "non-small cell lung carcinoma", and "pancreatitis". For the OMIM vocabulary, the keywords are: "workers", "metabolizing", and "increased susceptibility". We speculate that if there are any relevant biomedical keywords in common between two gene descriptions, then there are likely to be other keywords in common. Our conclusion from these experiments is that PDG-ACE is relatively robust to the vocabulary used.

**Table 3:** Vocabulary comparison. Hits for OMIM, NAÏVE and MeSH vocabularies.

		OMIM				NAIVE				MeSH			
LOCUS x	LOCUS	1	100	250	500	1	100	250	500	1	100	250	500
IL10	XPD	✔	✔			✔	✔		✔	✔	✔	✔	✔
GSTP1	COMT	✔				✔				✔	✔		
COMT	CCND1	✔	✔	✔	✔	✔				✔			
BARD1	XPD										✔		
CYP17	GADD45g												
TNFa	p27	✔				✔				✔			
BARD1	ESR1												
BARD1	p27												
GSTM1	CYP2e1	✔	✔			✔	✔	✔	✔	✔	✔	✔	
NR3C1	ESR2	✔				✔	✔	✔	✔	✔			
NR3C1	HDC												
RANK	TNFR2				✔						✔		
MAOA	SLC6A2	✔	✔	✔	✔	✔	✔	✔	✔	✔	✔	✔	✔
ALDH2	ADH1B	✔	✔	✔	✔	✔	✔	✔	✔	✔	✔	✔	✔
CD14	IL4Ra	✔	✔			✔	✔			✔			
CbetaS	MTHFR	✔				✔	✔			✔	✔	✔	
MTRR	MTHFR	✔	✔			✔	✔	✔	✔	✔	✔		✔
MTRR	FOLH1									✔			

### Case study

As an example of how PDG-ACE can aid in the understanding of complex disease etiology, we discuss its application. A recently published study [8] identified ten T2D-associated loci; five corresponding to genes previously associated with T2D, and five that had no prior association with T2D. Two of the loci are excluded, because one (rs9300039) is more than 1 MBP from the nearest annotated gene, and the other (rs8050136) is near the *FTO *gene, which is annotated as provisional in Entrez Gene and so was excluded by PDG-ACE. Using the remaining T2D-associated genes as input (*IGF2BP2*, *CDKAL1*, *CDKN2A*/*CDKN2B*, *PPARG*, *SLC30A8*, *HHEX*, *TCF7L2*, *KCNJ11*) we ran PDG-ACE with the MeSH vocabulary. We performed at least one million iterations for each test, and confirmed that each sample was sufficient, as described above. We searched up to +/-500 KBP from the transcription start site for each locus.

**Figure 3:**
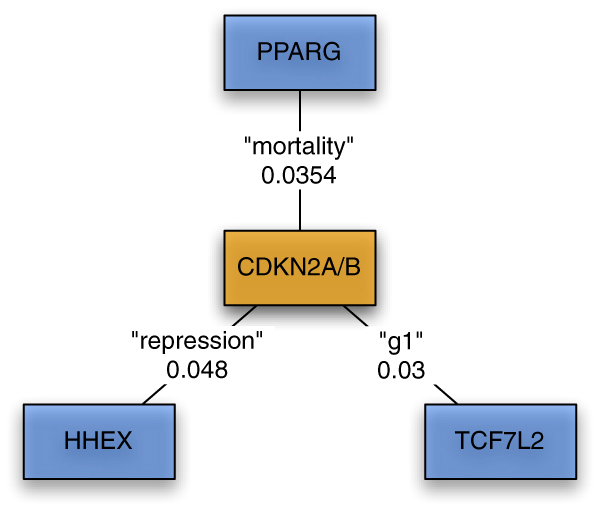
**Relationships discovered for FUSION genes**. PDG-ACE discovered relationships between CDKN2A/B and known T2DM genes from the FUSION study. Edge labels are keywords and their p-values.

As shown in Figure 3, PDG-ACE found significant commonality between the *CDKN2A*/*CDKN2B *locus and three other T2D candidate genes (*PPARG*, *HHEX*, and *TCF7L2*). No significant multi-gene effects were found for the *PPARG*-*HHEX*, *PPARG*-*TCF7L2*, and *HHEX*-*TCF7L2 *locus pairs. Notably, the *CDKN2A*/*B *locus was newly discovered by Scott, et al. [8], while all three of the genes related to *CDKN2A*/*B *by PDG-ACE were previously established as T2DM candidates. Here, PDG-ACE was able to fill in missing relationships among these genes.

The observation that the *CDKN2A*/*B *gene pair shows significant multi-gene effects with all three of these other T2D associated genes led us to the hypothesis that these genes form a cluster that may participate in a larger multi-gene effect that could be related to T2D susceptibility. To test this hypothesis, we used MetaCore from GeneGo, Inc. [20] to assess over-representation of the PDG-ACE identified gene set in Gene Ontology (GO) processes. Parameter settings used in GeneGo's "analyze networks" algorithm were to use only curated interactions, where the interactions included binding, direct/indirect, or unspecified types. GeneGo separates *CDKN2A *transcripts into two isoforms, *p14ARF *and *p16INK4*, yielding six entities. GeneGo finds that all six entities fit into the GO process GO:0050794, and the input set is significantly over-represented in this process, with a *p*-value < 0.01.

## Conclusion

The PDG-ACE algorithm takes a simplified approach to complex disease analysis. Assuming that multiple genetic influences converge on a single phenotype in complex diseases, PDG-ACE searches for common elements of text describing genes at disease-related loci, revealing potential underlying genetic influences on the phenotype of interest. Existing tools look for common elements of annotation among multiple genes including pathways, gene ontology, and expression. However, for most genes the annotation of these details is incomplete. The heuristic employed in PDG-ACE overcomes this shortcoming by using available text descriptions for genes, and is promising for generating hypotheses for genetic influences on complex disease. Clearly, however, PDG-ACE implements only an initial step in the refinement of such hypotheses, and other existing tools complement the approach.

We should also make note of possible limitations of PDG-ACE. The first is that it depends on descriptions that may not yet exist, and when they do may have a bias toward information garnered in studies of well-funded diseases. We believe that our experiments with different vocabularies indicate this bias is weak if there is any at all, but, clearly, are not conclusive. Another issue is that we make no attempt to identify the context of keywords computationally in order to decide equivalence of keywords. This has the advantage that the output is easy to understand, but also increases the false positive rate. We consider a keyword, common and significantly over-represented at a locus pair, to be a false positive if it is used in different contexts in the Entrez Gene records. Some subjectivity is involved in assessing the context of a keyword, but we informally estimate that 10% of keywords selected by PDG-ACE fall into this category. An additional challenge is in assessing a keyword that is clearly used in the same context across a locus pair, but the keyword cannot be placed into the context of the disease. These keywords may not be related to the disease or may reflect disease etiology that is not yet revealed by any other assessments.

## Competing interests

The authors declare that they have no competing interests.

## Authors' contributions

BK and RM co-developed the method, BK wrote the PDG-ACE software and utilities, and RM performed all experiments and analysis. Each contributed the corresponding sections to the manuscript, while BK was responsible for overall editing. Both authors have reviewed and approved the final manuscript.
